# Can follicular helper T cells be targeted to improve vaccine efficacy?

**DOI:** 10.12688/f1000research.7388.1

**Published:** 2016-01-20

**Authors:** Michelle A. Linterman, Danika L. Hill

**Affiliations:** 1 Laboratory of Lymphocyte Signalling and Development, Babraham Institute, Cambridge, CB22 3AT, UK

**Keywords:** T follicular helper cells, vaccines, immunity, antibody

## Abstract

The success of most vaccines relies on the generation of antibodies to provide protection against subsequent infection; this in turn depends on a robust germinal centre (GC) response that culminates in the production of long-lived antibody-secreting plasma cells. The size and quality of the GC response are directed by a specialised subset of CD4 ^+^ T cells: T follicular helper (Tfh) cells. Tfh cells provide growth and differentiation signals to GC B cells and mediate positive selection of high-affinity B cell clones in the GC, thereby determining which B cells exit the GC as plasma cells and memory B cells. Because of their central role in the production of long-lasting humoral immunity, Tfh cells represent an interesting target for rational vaccine design.

## Introduction

Vaccination is one of the most successful, cost-effective interventions for combating infectious disease, thereby reducing infection-related disease, disability and death worldwide ^1^. Despite this enormous success, there are still multiple infections that require a vaccination solution, including vaccines that protect against HIV and malaria ^2, 3^, and a way to improve vaccine efficacy in older persons ^4^. The majority of current vaccines have been developed empirically rather than rationally, suggesting that a change in approach to vaccine development may enable breakthroughs in vaccine design ^5^. All routine human vaccinations, with the exception of the Bacillus Calmette-Guérin tuberculosis vaccine, provide protection by generating antibodies that block the ability of a pathogen to establish an infection and that target it for destruction. Vaccine-induced antibody responses are supported by T follicular helper (Tfh) cells; here, we discuss how advances in the knowledge of Tfh cell biology could be used to improve vaccine efficacy.

The production of vaccine-specific antibodies can occur via two cellular routes: the extrafollicular or germinal centre (GC) responses. The extrafollicular response produces an initial burst of antibodies early after immunisation and can occur with or without T cell help ^6^. These extrafollicular plasma cells are short-lived and because of this are not able to provide a long-term source of protective antibodies ^7^. The GC is a specialised microenvironment that forms in secondary lymphoid tissues after immunisation when antigen-activated B cells migrate to the B cell follicle, begin to proliferate, and undergo somatic hypermutation (SHM) of their immunoglobulin genes ^8^. The cellular products of the GC are long-lived plasma and memory B cells that can provide protection for decades after initial exposure ^9^. Because of the longevity of these cells, the GC represents an exciting target to improve vaccine responses in situations in which there is an unmet clinical need.

**Figure 1.**
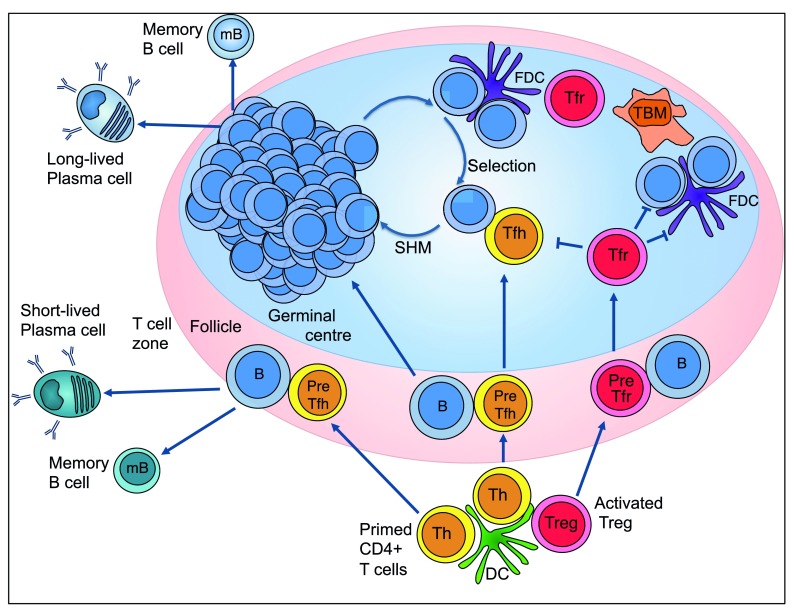
Pathways to antibody production. The germinal centre (GC) is initiated when activated B cells migrate into the B cell follicle, begin to divide, and mutate their B-cell receptor (BCR) genes. These proliferating centroblasts then exit the cell cycle, becoming centrocytes that use their mutated BCR to collect antigen from the surface of follicular dendritic cells (FDCs) and present processed peptide on major histocompatibility complex class II to T follicular helper (Tfh) cells. Tfh and T follicular regulatory (Tfr) cell differentiation initiates during T cell priming when Bcl-6, the transcription factor required for Tfh and Tfr cell differentiation, is upregulated. Subsequent interactions with B cells stabilise Bcl-6 expression in pre-Tfh and pre-Tfr cells, allowing them to migrate into the GC where Tfh cells provide help to centrocytes and Tfr cells act as GC suppressors. As an alternative to entering the GC, antigen-activated B cells can also receive T cell help and differentiate into short-lived extrafollicular plasma cells that produce the first wave of protective antibodies. DC, dendritic cell; FDC, follicular dendritic cell; mB, memory B cell; SHM, somatic hypermutation; TBM, tingible body macrophage; Th, T helper cell; Treg, regulatory T cell.

The GC is a microenvironment of intense cellular collaboration: GC B cells, Tfh cells, T follicular regulatory (Tfr) cells, tingible body macrophages and follicular dendritic cells (FDCs) act together to generate a robust response ( Figure 1). The “multi-player” nature of the GC means that there are a number of cellular targets that can be manipulated in the GC during vaccination in an attempt to modulate its output. Here, we will discuss whether targeting Tfh and Tfr cells may be a successful strategy for improving the GC in response to vaccination.

## Follicular T cells and the GC response

In physiological conditions, the GC is absolutely dependent on T cell help for its formation. Experimentally, immunisation with superantigens can initiate GC formation in the absence of T cells, however, the response collapses after five days, demonstrating the absolute requirement of T cells for the GC to produce plasma cells ^10^. The specific requirement for T cell help is fulfilled by a specialised subset of CD4 ^+^ helper T cells: Tfh cells. Tfh cells differentiate in response to immunisation by using a specific differentiation pathway that requires various cytokine signals and multiple rounds of antigen presentation that culminates in the expression of the transcriptional repressor Bcl-6 and localisation to the GC ^11^. The signals that are required for Tfh cell development have been well characterised in both humans and mice; these are summarised in Table 1 ^12– 42^. These pathways may represent viable targets to manipulate the number of Tfh cells that form after vaccination.

**Table 1.** Several surface receptors play vital roles in regulating T follicular helper development by incorporating signals that result from interactions with a variety of cell types.

Surface receptor	Interacting cell type	Downstream signalling	Mouse Tfh	Human Tfh	References
ICOS	DC, B cell	PI3K/Akt	↑	↑	12– 16
CD28	DC, B cell	PI3K	↑	ND	17– 20
OX40	DC	PI3K/NFkB	↑	↑	21– 23
CD84/Ly108	B cell	SAP	↑	↑	24– 27
PD1	DC, B cell	SHP2	↑	ND	28– 30
IL-21R	T cell	STAT1/STAT3	↑	=	16, 31, 32
IL-6R	DC, B cell	STAT1/STAT3	↑	↑	33– 35
IL-12R/IL-23R	DC	STAT4	ND	↑	36, 37
TGF-BR I and II	DC	STAT4/STAT3	↓	↑	38, 39
IL-2R	T cell	STAT5	↓	ND	40, 41
IL-10R	DC, B cell	STAT3	↓	↑	16, 42

For each receptor expressed by T follicular helper (Tfh) cells or their precursors, the predominant downstream signalling pathway is indicated. Surface receptors and signals were deemed to influence Tfh cell development if Tfh cell frequencies were altered in deficient mice or in human patients with genetic deficiency. ↑, an increase in T follicular helper frequencies; ↓, a decrease in T follicular helper frequencies; =, no change in T follicular helper frequencies; DC, dendritic cell; ND, not determined.

After establishment of the GC, it divides into two distinct zones: the GC B cells rapidly divide in the dark zone and undergo SHM of their B-cell receptor (BCR) genes and then exit the cell cycle and migrate to the light zone of the GC, where the fully differentiated Tfh cells are located. The random nature of SHM requires that mutated B cells undergo a selection process before they exit the GC as plasma cells or memory B cells. The GC B cells test their newly mutated BCR by collecting antigen-containing immune complexes from the surface of FDCs and then present processed antigen to Tfh cells on major histocompatibility complex (MHC) class II ^43^. The B cells that are able to present the most antigen to Tfh cells are the recipients of T cell help ^44^, which results in the upregulation of c-myc and subsequent return to the dark zone to undergo further rounds of proliferation and mutation ^45– 47^. By this mechanism, Tfh cells act as a limiting factor in the selection of high-affinity GC B cells. This could simply be numerical, as B cells outnumber Tfh cells in the GC. Alternatively, there may be an interaction threshold that needs to be overcome before Tfh and B cells form productive conjugates in the GC. During Tfh and B cell interactions in the GC, there is a bidirectional exchange of signals: Tfh cells provide help in the form of CD40L, interleukin-21 (IL-21) and IL-4 to GC B cells, which supports proliferation and survival, while B cells provide inducible T cell co-stimulator ligand (ICOSL) to Tfh cells ^48, 49^. Thus, Tfh cells facilitate the preferential expansion and mutation of high-affinity GC B cell clones and are key regulators of the size and quality of the GC response.

In addition to Tfh cells, there is a second specialised subset of CD4 ^+^ T cells present in the GC: Tfr cells. Tfr cells derive from Foxp3 ^+^ regulatory T (Treg) cells and act as suppressors of the GC response. Tfr cells limit the size of the GC response, thereby acting as a counterbalance to Tfh cells ^50– 52^. Although there are some transcriptional and phenotypic similarities between Tfh and Tfr cells, functionally Tfr cells are suppressive and thus resemble conventional Treg cells ^53^.

Together, Tfh and Tfr cells are key regulators of the GC response; Tfh cells positively control the size and output of GC, whereas Tfr cells act as negative regulators of the response. This suggests that strategies to enhance Tfh number or function (or both) or reduce Tfr cells may enhance GC responses and promote a more potent response to vaccination.

## Circulating peripheral blood Tfh as biomarkers of GC Tfh cells

The majority of advances in Tfh and Tfr cell biology have occurred through studying secondary lymphoid tissues in mice. This is largely because of the impressive range of tools (e.g., genetically modified mice) that allow precise dissection of GC biology in mice and because access to healthy human lymphoid tissue can be difficult, particularly for the purpose of studying GC responses to a defined antigenic stimulus. In an attempt to circumvent this issue, a circulating cellular biomarker of GC Tfh cells has been used to further investigate Tfh cell biology in humans. These cells have been coined circulating Tfh-like (cTfh) cells and were first identified in *sanroque* mice and in patients with systemic lupus erythematous and were defined by CXCR5, programmed cell death protein 1 (PD-1), and ICOS expression ^54^. Subsequently, it has been shown that an increased frequency of cTfh cells coincides with the peak GC response in mice and the plasmablast response to influenza vaccination in humans ^55, 56^. These studies suggest that cTfh cells may be a key tool for studying the role of Tfh cells in human vaccine responses. However, the use of cTfh cells as a surrogate of GC Tfh cell responses in humans requires a robust assessment of the strengths and limitations of this approach.

Studies in both humans and mice support a link between the GC Tfh and cTfh cells. Human cTfh cells can provide help to B cells *in vitro* and upon stimulation display several features consistent with GC Tfh cells, including ICOS expression and expression of IL-21 and CXCL13 ^57– 59^. Although cTfh cells do not express BCL6, they have low levels of BLIMP1 and express cMAF, and this indicates that they share features of transcriptional control with GC Tfh cells ^57– 59^. Several human immunodeficiency syndromes that are associated with severely impaired GC responses due to loss of functional CD40L ^60^, ICOS ^15, 61^, STAT3 ^62^ or IL-12βR1 ^36^ display corresponding reductions in blood cTfh cells, suggesting that cTfh cells can be a biomarker for an active GC response. Conversely, mice deficient for *Sh2d1a* have impaired GC reactions but unchanged cTfh frequencies ^55^. Consistent with this, patients with X-linked lymphoproliferative disease (XLP) caused by defects in *SH2D1A*, or healthy XLP carriers, did not display alterations in cTfh frequencies ^55^. These data suggest that although cTfh cells resemble GC Tfh cells, a GC reaction is not required for cTfh cell development, which parallels the development of extrafollicular Tfh cells ^63^. One possibility is that cTfh cells are memory cells that are induced upon vaccination to enable fast GC Tfh responses following subsequent infection. Consistent with this idea, tetanus- and smallpox-specific cTfh cells can be identified in humans years after vaccination ^58, 64^. In mice, cTfh cells have the capacity to become GC Tfh cells and support the GC response ^55, 65^, suggesting that cTfh cells may be an important component of secondary immune responses and therefore a biologically relevant cell population in successful vaccination. Despite the recent surge in correlative studies assessing cTfh cells in a multitude of disease settings, unsupervised comparisons of gene expression in GC Tfh cells have not been performed in blood and lymphoid tissue samples from the same individual ^58^, and antigen-specific responses have not been determined. Addressing these issues will help to clarify the relationship between circulating and GC Tfh cells.

An interesting feature of GC Tfh cells is their well-described heterogeneity ^66^, and cTfh cells are not an exception. Analysis of blood CD4 ^+^CXCR5 ^+^ cells for expression of PD1, CCR7, CXCR3, CCR6 and ICOS has been proposed to define nine populations of cTfh cells ^67^. However, across the range of studies, robust B cell helper function *in vitro* has consistently been demonstrated for CD4 ^+^CXCR5 ^+^ cells that express high levels of PD-1 or ICOS or both ^67^. CXCR3 and CCR6 expression on cTfh enables identification of cTfh cells with Th1-like (cTfh1, CXCR3 ^+^CCR6 ^−^), Th2-like (cTfh2, CXCR3 ^−^CCR6 ^−^) and Th17-like (cTfh17, CXCR3 ^−^CCR6 ^+^) properties, including the expression of transcription factors and cytokines that define these T helper subsets ^57^. cTfh2 and cTfh17 can support naïve and memory B cells to produce antibodies *in vitro*, whereas cTfh1 cells have limited *in vitro* helper function ^57, 58^, although following influenza vaccination a population of ICOS ^+^ cTfh1 cells were able to help memory B cells make antibodies ^56^. One limitation of these studies is that it remains unclear to what extent *in vitro* B cell helper function reflects effective GC Tfh help *in vivo.* Although these cTfh cell subtypes have been identified in blood, characterisation of GC Tfh cell populations by using these markers has been limited, calling into question the relevance of these subsets to GC biology. However, tonsillar Tfh can co-express BCL6 and RORγt ^67^ and a proportion of human lymph node Tfh cells express CXCR3 (D.L. Hill, unpublished), and this suggests that comparable heterogeneity exists within in the GC Tfh cell population. But whether there is a specialised role for Th1/Th2/Th17 polarised GC Tfh cells in the GC has yet to be elucidated.

The polarisation of GC Tfh cells depends on the stimuli provided during differentiation. In mice, Th2-biased infections produce IL-4-secreting GC Tfh cells, whereas Th1-biased infections support interferon-gamma-positive (IFNγ ^+^) GC Tfh cells ^68– 71^. In humans, cTfh2 cell frequency increases in people with Th2-polarised *Schistosoma japonicum* infection ^72^, whereas cTfh1 cells are preferentially expanded during Th1-biased acute *Plasmodium falciparum* infection and after seasonal influenza vaccination ^56, 73^. Thus, different cytokine environments induced by specific infections or immunisations appear to drive Tfh cell polarisation and may enable Tfh cells to appropriately support B cell production of the antibody isotype required to clear the infection. For example, in mice, IFNγ ^+^ Tfh cells could be found in conjugates with Ig2a ^+^ B cells, whereas IL-4 ^+^ Tfh cells were more likely to be paired with IgG1 ^+^ B cells ^74^. Immunity against pathogens relies upon production of specific antibody isotypes that ultimately play an important role in clearing infections. For example, inappropriate production of Th1-supported isotypes to the parasitic roundworm *Wuchereria bancrofti* ^75^ and Th2-supported isotypes in *P. falciparum* malaria ^76^ correlates with poor disease outcomes. Therefore, cTfh cell heterogeneity may reflect the ability of Tfh cells to be shaped by the environmental signals present during differentiation, which enables them to guide an appropriate B cell response to infection or vaccination, to facilitate pathogen clearance.

**Figure 2.**
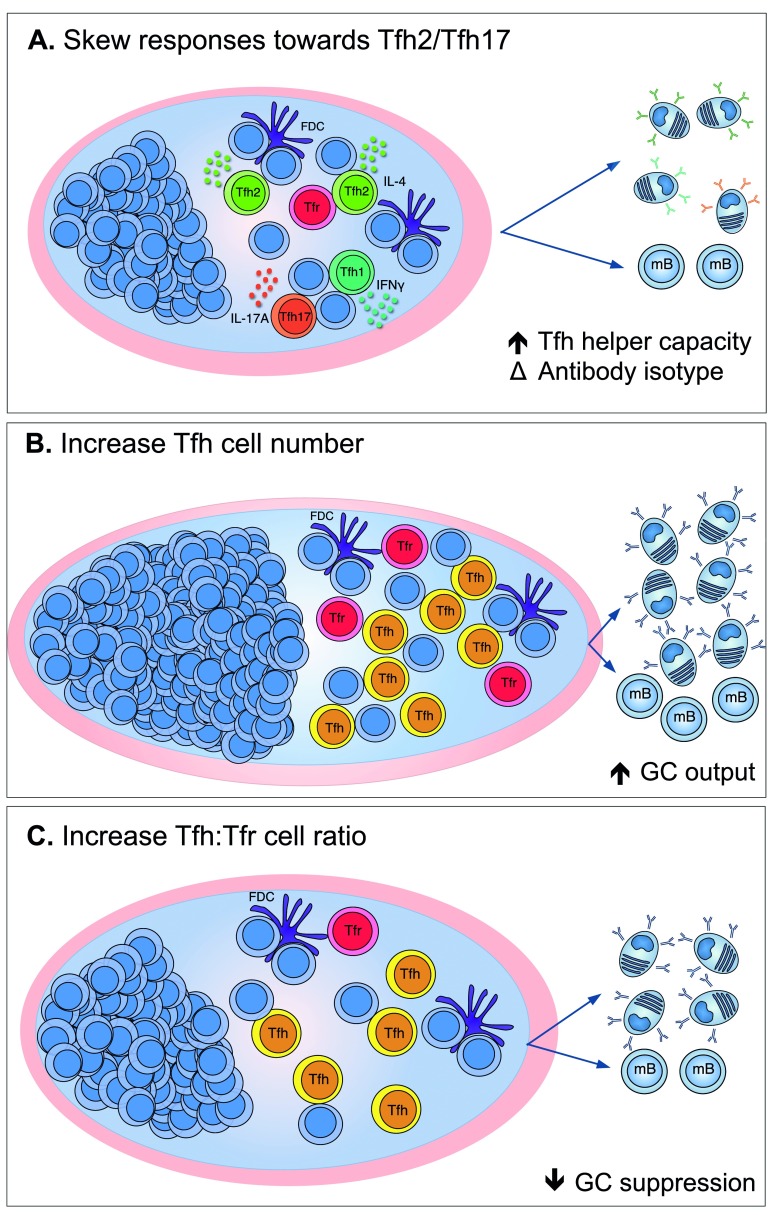
Strategies for manipulating follicular T cells to enhance the output of the germinal centre (GC) response. ( **A**) Altering the balance of different subsets of T follicular helper (Tfh) cells toward Tfh2 and Tfh17 cells may enhance the B cell helper capacity of the Tfh cells within the GC and the production of the necessary antibody isotypes. ( **B**) Increasing the number of Tfh cells may help increase the size and output of the GC response. ( **C**) Reducing the frequency of suppressive T follicular regulatory (Tfr) cells may enhance the output of the GC response. FDC, follicular dendritic cell; IFNγ, interferon-gamma; IL-17A, interleukin-17A; mB, memory B cell.

It has been proposed that the limited efficacy of seasonal influenza vaccination results from the preferential induction of cTfh1 cells ^58^. As such, skewing Tfh cells away from Tfh1-like and toward Tfh2/17-like may represent a potential target to enhance antibody titres following influenza vaccination ( Figure 2A). Interestingly, blocking the Th1 cytokines IL-2 and tumour necrosis factor (TNF) improved Tfh-mediated B cell help *in vitro* ^77, 78^. However, this approach may not be effective for generating protective responses to vaccination *in vivo*. Passive transfer of broadly neutralising antibodies to hemagglutinin can protect mice from succumbing to experimental influenza infection. Importantly, for some clones, this protection is conferred only by a Th1 polarised IgG2a antibody and not Th2 polarised IgG1, despite having the same ability to bind hemagglutinin ^79^. This suggests that production of Th1-supported isotypes and the selective induction of Th1-like Tfh cells are likely important for generating protective influenza vaccine responses. Yet as current influenza vaccine formulations fail to generate a protective immune response in up to 30% of vaccine recipients ^80^, further enhancing Tfh cell responses may improve vaccine efficacy.

The potential use of cTfh cell subsets as a biomarker of vaccine efficacy is an attractive possibility that would be easily amenable to clinical trials. Although further study is needed, mounting evidence supports cTfh cells as a relevant population for the study of vaccine responses in humans. However, it is clear that there are limitations for using cTfh cells to study the GC Tfh cell response, as although they appear to be a biomarker for the GC response, they differ from bona fide GC Tfh cells in a number of aspects, including BCL6 expression and dependence on SAP for their differentiation. Because of this, analysing cTfh cells will likely not become a substitute for studies aimed at understanding GC Tfh cell biology, but rather represents an additional tool to interrogate the human response to vaccination. Research that combines assessment of cTfh cells after vaccination, GC Tfh cells from human secondary lymphoid tissues and mouse vaccination models will enable thorough interrogation of strategies that target Tfh cells to improve vaccine efficacy.

## Potential avenues for modifying follicular T cells to enhance vaccination efficacy

Within the GC, Tfh cells support the GC response and Tfr cells negatively regulate the magnitude of the GC. As such, manipulating the frequency of Tfh cells or enhancing their function may improve the GC response. Conversely, reducing the number of Tfr cells or their functional capacity may also increase antibody responses to vaccination. Here, we explore the potential strategies for manipulating these T cell subsets with the view to increase GC output following vaccination.

### Increasing the frequency of Tfh cells

The numbers of Tfh cells and GC B cells positively correlate ^81, 82^, as do the numbers of cTfh and blood plasmablasts after influenza vaccination in humans ^56^. This suggests that strategies to augment Tfh cell number may be a rational approach to enhance vaccine responses ( Figure 2B).

Antigen presentation and recognition are central to Tfh cell differentiation, and hence providing ample antigen may enhance Tfh-driven vaccine responses. Increasing the dose of protein antigen enhances the magnitude of Tfh cell responses in mice ^82^, and in older people a higher dose of seasonal influenza vaccine increases neutralising antibody titres ^83, 84^. This increased antigen availability may have enabled increased peptide-MHC II presentation to T cells, resulting in enhanced Tfh cell differentiation and function. Specifically targeting antigen to the CD8 ^+^ DC receptor Clec9A enhanced MHC II presentation, antibody responses, Tfh cell numbers and memory B cells in the absence of adjuvants in mice and non-human primates ^85– 87^. Likewise, when B cells present high levels of peptide-MHC II to Tfh cells, they are able to gain entry to the GC and, once within the GC, are more likely to be maintained ^88, 89^. Consistent with this, the ongoing presence of antigen in people with chronic HIV and hepatitis B virus is associated with expansions in cTfh cells ^90, 91^. This suggests that increasing antigen availability may be a strategy to enhance Tfh cell numbers in response to vaccination. However, despite elevated cTfh cells, the majority of chronically HIV-infected individuals fail to mount broadly neutralising antibody responses ^92^. Furthermore, in mice, increasing Tfh cell number by provision of soluble peptide did not increase the frequency of high-affinity B cells in the early phase of the vaccine response ^93^. This suggests that, in some cases, solely increasing Tfh numbers may not be sufficient to enhance vaccine efficacy, and approaches may need to be tailored to the specific vaccination challenge.

One of the potential challenges to specifically targeting Tfh cells during vaccination is to not perturb normal immune cell homeostasis. The best way to do this has long been considered “the immunologist’s dirty little secret”—adjuvants, which trigger T and B cells to respond to antigen ^94^. Currently, only a handful of different adjuvants have been used in licensed vaccines ^95^, and the use of Alum is the most widespread. Novel or modified adjuvants may prove to be an effective strategy to skew helper T cells to differentiate toward the Tfh cell subset and promote GC responses. The squalene adjuvant MF59 has been shown to increase the quantity, diversity and affinity of antibodies produced following pandemic influenza vaccination ^96– 98^. MF59 increases GC B cells, Tfh cells, and antigen-specific DCs following immunisation in mice ^99, 100^, and this suggests that MF59 may act via DCs to enhance the GC response. The use of Toll-like receptor (TLR) agonists as adjuvants has been successful in enhancing vaccine responses in mice and non-human primates. Nanoparticles containing TLR4 and TLR7/8 agonists prolonged GC reactions, improved antibody quality, supported memory B cell development in mice ^101, 102^, and protected against secondary influenza infection ^101^. It would be particularly pertinent to consider adjuvants that trigger pathways known to enhance Tfh cell differentiation ( Table 1). For example, immunisations supplemented with TLR9 agonists enhanced antibody responses and Tfh and GC B cell numbers in mice via DC production of the Tfh-promoting cytokine, IL-6 ^33, 103^. Alternatively, ICOSL binding is a requisite event in multiple stages of Tfh cell development but is not an essential requirement for Th1 or Th2 cell differentiation ^13, 104, 105^. Upregulation of ICOSL on DCs upon the addition of a TLR2 agonist correlated with enhanced antibody production following protein immunisation *in vivo* ^106^. Thus, TLR signalling in DCs and B cells could be specifically directed to enhance vaccine antibody and Tfh cell responses.

Another potential strategy to enhance Tfh cell numbers is to use adjuvants to modulate the cytokines produced by antigen-presenting cells to promote Tfh cell differentiation. In mice, IL-6 and IL-21 support Tfh cell differentiation, whereas IL-2 suppresses Tfh cell fate ^31, 32, 35, 40, 107^. In humans, an entirely separate cohort of cytokines support Tfh cells: IL-12, IL-23 and transforming growth factor-beta (TGF-β) ( Table 1) ^36– 38^. Because of this, the IL-12/STAT4 axis may be a potential target to enhance Tfh cells in humans. However, preclinical trials of an IL-12 expression plasmid adjuvant did not enhance vaccine antibody responses ^108, 109^. Alternatively, the addition of Fc-fused IL-7 enhanced B cell and Tfh responses to influenza vaccination in mice and cynomolgus monkeys ^110^, suggesting that delivery of a generic T cell survival signal could be sufficient to enhance the vaccination responses. The different cytokine requirements between mice and humans for Tfh cell differentiation demonstrate the importance of studying human Tfh cell biology.

### Altering Tfh cell function

The pathways involved in Tfh development are well established ( Table 1); however, the signals that regulate Tfh cell effector function are less well described. Increased antigen presentation from B cells increases Tfh cell production of the cytokines IL-4 and IL-21 ^48^. These observations suggest that increasing vaccine antigen dose or targeting antigen to B cells may improve Tfh function and the quality of the GC reaction. ICOSL expression by GC B cells promoted calcium-dependent CD40L expression from Tfh cells, and this feed-forward signalling loop provided a competitive advantage to ICOSL-expressing B cells ^49^. Because help via CD40L, IL-21 and IL-4 is important for GC B cell-positive selection, it raises the possibility that adjuvant approaches that lead to increased ICOSL or CD40L in GC B and Tfh cells, respectively, could be a strategy to enhance vaccine responses.

### Diminishing suppression of the GC by Tfr cells

A reduction in the frequency of Tfr cells may be a useful approach to enhance the GC response, particularly in situations such as ageing in which an increased number of Tfr cells correlates with a smaller GC response ^111^. In mice, it is possible to alter the ratio of Tfh to Tfr cells simply by using different adjuvants; the more the ratio favours Tfh cells, the larger the GC response ( Figure 2C) ^103, 112^. To specifically manipulate Tfr cells, two key inhibitory molecules may be potential targets: cytotoxic T-lymphocyte-associated protein 4 (CTLA-4) and PD-1. CTLA-4 is a suppressive mechanism by which Treg and Tfr cells can control GC response to vaccination through limiting CD28 signalling that is important for Tfh maintenance ^17, 113, 114^. Inhibiting this receptor in mice increases the number of antigen-specific Tfh cells, plasma and memory B cells following immunisation. Proof-of-principle testing could be performed in melanoma patients receiving the CTLA-4 inhibitor ipilimumab and vaccination, although the side effects and cost associated with this compound would prohibit its use with routine vaccines. Another interesting target is PD-1, as signalling through this receptor limits Tfr cell differentiation in mice ^30^. However, this is likely not to be practicable as PD-1 is also expressed highly on Tfh cells ^115^ and targeting PD-1 on Tfr cells specifically would be difficult. Also, some adjuvants support the generation of induced Tfr cells via a PD-L1-dependent mechanism ^112^, suggesting that the role for PD-1 signalling for Tfr cell formation is context-dependent and more complex than originally thought. The main barrier for determining whether inhibiting Tfr cells is a logical strategy to improve vaccination is our lack of knowledge of their precise role in the GC, particularly whether they suppress humoral autoimmunity arising from the GC ^53^. Currently, there are conflicting reports about whether Tfr cells constrain vaccine-specific responses or non-vaccine-specific responses in the GC ^50– 52^. For these cells to be a viable target, definitive evidence would be needed to demonstrate that the role of Tfr cells in the GC is to restrain responses to foreign, rather than self, antigens.

## Potential dangers of enhancing Tfh responses

Although it is clear that Tfh cells are essential for a productive response to vaccination, they have also been implicated in a number of autoimmune conditions as key drivers of disease. *Sanroque* mice have a point mutation in the *Roquin1* gene ( *Rc3h1*) that causes a lupus-like phenotype that is driven by Tfh cells that support GCs in the absence of exogenous antigen ^116, 117^. As B cells can acquire self-reactivity during somatic hyper-mutation, findings from the *sanroque* mice suggest that increases in Tfh cell number may lead to a break in GC tolerance, enabling self-reactive B cells to be selected in the GC. Correspondingly, several autoimmune conditions have been associated with an increase in cTfh frequencies ^54, 118– 120^ or have a skewed cTfh population away from cTfh1 and toward cTfh2 or cTfh17 or both ^57, 118, 121, 122^. This is an important consideration in the context of enhancing Tfh responses to vaccination, particularly in older persons, as the occurrence of autoantibodies increases with age ^123^, suggesting that the B cell pool may contain a higher frequency of autoreactive B cells able to enter the GC. Taken together, these studies demonstrate a potential hazard of increasing Tfh cell frequencies or function in vaccination and indicate that autoantibody production would need to be accounted for in future study design as a possible outcome.

## Concluding remarks

The GC is critical for the production of long-lived antibody-secreting plasma cells after vaccination, making it a promising cellular response to improve vaccine efficacy. There are many players in the GC response; Tfh and Tfr cells tightly control its size and output and thus make them key targets to manipulate in vaccine design. Altering vaccines in a way that increases Tfh cell formation or function (or both) or reduces the suppression exerted on the GC by Tfr cells may be a rational strategy to improve vaccine responses. As vaccines need to have an extremely high safety profile, any perturbations to vaccines must be very low-risk. Because of this, the most logical way to manipulate follicular T cells is to use antigen doses or adjuvants that favour differentiation of Tfh cells with excellent B cell helper capacity, and suppress Tfr cell development. Recent research into next-generation adjuvants demonstrates that adjuvants that support enhanced antibody production also associate with increased numbers of Tfh cell in experimental animals. To determine whether this can translate into enhanced Tfh responses in humans, cTfh cells will be a useful biomarker of GC Tfh responses in preliminary clinical trials. Further research in both humans and animal models into precisely how to manipulate Tfh and Tfr cells to improve vaccine responses may enable us to address some of our current unmet clinical requirements for improved vaccines.
